# Bits per Spike as a Betting Game: An interpretable unit for held-out log-likelihood in neural data analysis

**Published:** 2026-08-28

**Authors:** Alex H. Williams

**Affiliations:** Center for Neural Science, New York University; Center for Computational Neuroscience, Flatiron Institute

**Keywords:** bits per spike, held-out log-likelihood, e-values, anytime-valid inference, game-theoretic statistics, generalized linear models, spike trains

## Abstract

Held-out log-likelihood is the standard currency for comparing statistical models of neural spike trains, and is often reported as *bits per spike* relative to a homogeneous Poisson baseline. The units of this metric are difficult to reason about: it is rarely obvious whether an improvement of, say, 0.34 bits per spike is a large effect or a negligible one. This note develops an interpretation of held-out log-likelihood borrowed from game-theoretic statistics. If ℒ denotes a model’s expected log-likelihood ratio to the homogeneous Poisson baseline, then we show that -log(α)/ℒ represents the number of held-out time bins of recording needed to reject the baseline at level α under a particular null hypothesis testing procedure based on a betting game. Thus, a simple re-scaling of the heldout log-likelihood yields an intuitive metric of model performance in units of recording time which answers “how much heldout data would I need, on average, to disprove the null.” We illustrate the construction on head-direction cells recorded in mouse anterior thalamus, where a generalized linear model reaches significance in roughly 120 ms of held-out data for a strongly tuned cell and roughly 11 s for a moderately tuned cell.

Whenever we fit a model to neural or behavioral data, we need to benchmark it against simpler or well-known baselines. Typically this is done by reporting the difference in log-likelihoods on heldout data. For example, the popular “bits per spike” performance metric (see e.g. Pillow et al. 2008) is simply the log (base 2) likelihood of the model minus the log (base 2) likelihood of a homogeneous Poisson process (or another appropriate baseline model), divided by the total number of spikes in the dataset.

This note offers some thoughts on how we can interpret this performance measure. For example, if my model gives a 0.34 bits per spike improvement over the baseline, should I interpret that as very good? Marginal? Completely inconsequential?

This note will focus on a particular interpretation that imagines the model playing a betting game against the “market” defined by the baseline model. This game-theoretic framing has gained traction in a certain corner of statistics (see the references for a brief list of introductory resources).

## 1 Basic Setup

Suppose that we have fit a model Q and a baseline B on training data and that we’re now ready to compare them head-to-head on heldout test data. Let $X_{1},X_{2},X_{3},\ldots$ denote a (potentially infinite) sequence of heldout data samples. We assume these samples are independent and identically distributed according to an unknown distribution P. Our hope is that Q is in some sense “closer” to the true distribution P than the baseline model B.

For simplicity, we’ll assume that Q and B have strictly positive probability density or mass functions q(x)>0 and b(x)>0. This allows us to compute *likelihood ratios*, q(X)/b(X) for X∼P, without having to worry about divide-by-zero errors.^1^

A nice measure of performance is the expected log-likelihood ratio:

(1) $$ℒ=E_{X∼P}[log\;q(X)/b(X)]=E_{X∼P}[log\;q(X)-log\;b(X)]$$

Note that the expectation is computed under P. Since P is unknown in real world situations, we can estimate the expression above by holding out a test set with T data samples and approximating the expectation with an empirical average:

(2) $$ℒ\approx \hat{ℒ}=\frac{1}{T}\sum_{t=1}^{T}log\;q\left(X_{t}\right)/b\left(X_{t}\right)=\frac{1}{T}\sum_{t=1}^{T}[log\;q\left(X_{t}\right)-log\;b\left(X_{t}\right)]$$

We have thus far used natural logarithms, but substituting base-2 logarithms can aid interpretation. By the change of base formula, ${ℒ}_{2}=ℒ/\;log(2)$ is the expected base-2 log likelihood. The base-2 log likelihood has units of “bits”, and normalizing by the number of spikes gives the popular “bits per spike” metric.

Even more intriguingly, we will be able to draw a connection to null hypothesis testing at significance threshold α (for example, α=0.05). It turns out that the reciprocal of the expected base-(1/α) log likelihood, ${ℒ}_{1/\alpha }^{-1}=-log(\alpha )/ℒ$, will have a very satisfying interpretation as the number of heldout data samples needed to reject the baseline model as a null hypothesis.

*You don’t need to understand the betting game to use this result!* If you wish to skip the derivation, you can jump straight to the Take Home Message at the end of this note.

## 2 A Demo of the Betting Game

Our goal is to come up with intuitive interpretations of ℒ as a measure of model performance. One way to approach this is to imagine model Q as a “player” in a betting game. On each round of the game, we sample a heldout datapoint X∼P and pay the player based on how well they predicted the outcome. Each betting contract comes at a price, which is determined by the baseline model B. Intuitively, the more that Q is able to “beat the market” by accumulating wealth, the stronger the evidence we have in favor of Q over B.

**Figure 1.**
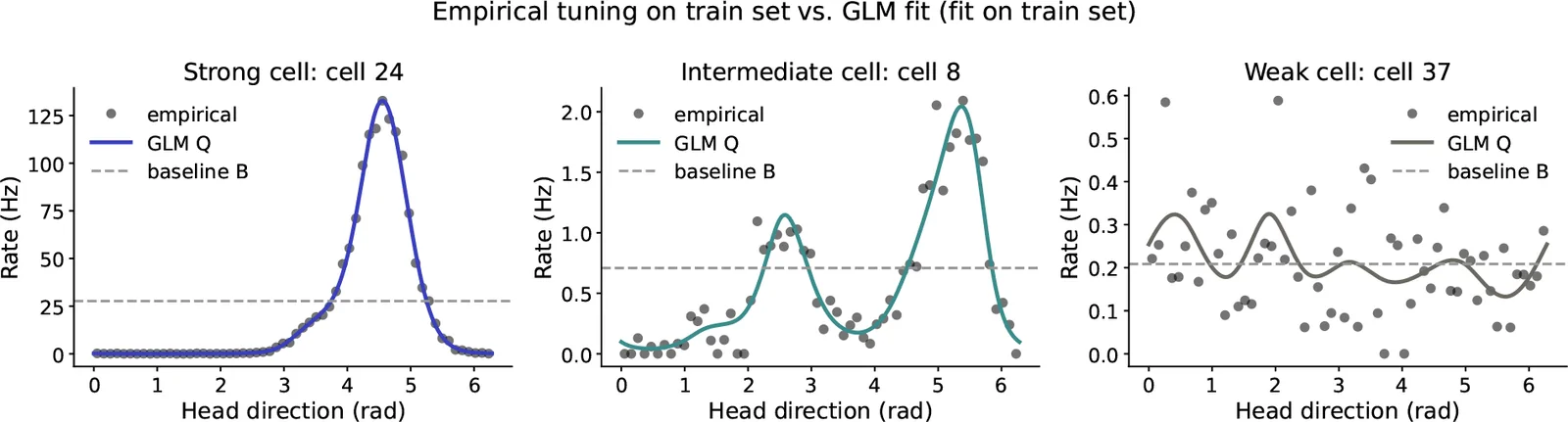
Three example cells with strong, intermediate, and weak head direction modulation. For each cell we fit a GLM model, Q, and a flat baseline model, B.

Before we get to the precise details of the game, let’s look at a few examples. Figure 1 shows tuning curves fit to three example head-direction cells from the mouse anterior thalamus (Peyrache et al. 2015), distributed through the **nemos** (NeMoS Developers 2025) library. Each cell’s spiking is modulated by the animal’s current head direction, and binning spike counts by head-direction angle gives *empirical firing rates* (grey dots). We then discretized spike trains using Δ = 0.05 second time bins and used **nemos** to fit a GLM model with cyclic B-spline basis functions (solid line, Q), and a homogeneous Poisson process as a baseline model (dashed grey line).

Concretely, model Q is a Poisson GLM whose single covariate is the animal’s head direction in each bin, expanded in ten cyclic B-spline basis functions and fit by unregularized maximum likelihood with L-BFGS. The baseline B is a homogeneous Poisson process whose rate is the mean spike count per bin, estimated on the same training half. Each cell’s recording was split 50/50 into training and test halves after shuffling the order of time bins, and the whole procedure was repeated for ten independent random splits. The three cells were selected by their degree of head-direction modulation: the most strongly modulated unit, a unit at the median of the modulation distribution, and the least modulated unit subject to a floor of 200 spikes.

The plots above were generated using 50% of the observations as “training data”. The remaining 50% of the observations serve as heldout “test data” which we use below to play the forecasting game.

In the game, player Q gambles their wealth over discrete rounds of betting. In each round, the player forecasts and places a bet on the number of spikes they’ll see in the next time bin. Intuitively, if Q is a much better model of the data than B then the player has an “edge” that they can exploit and generate returns very quickly—in fact, it turns out to be exponentially fast.

**Figure 2.**
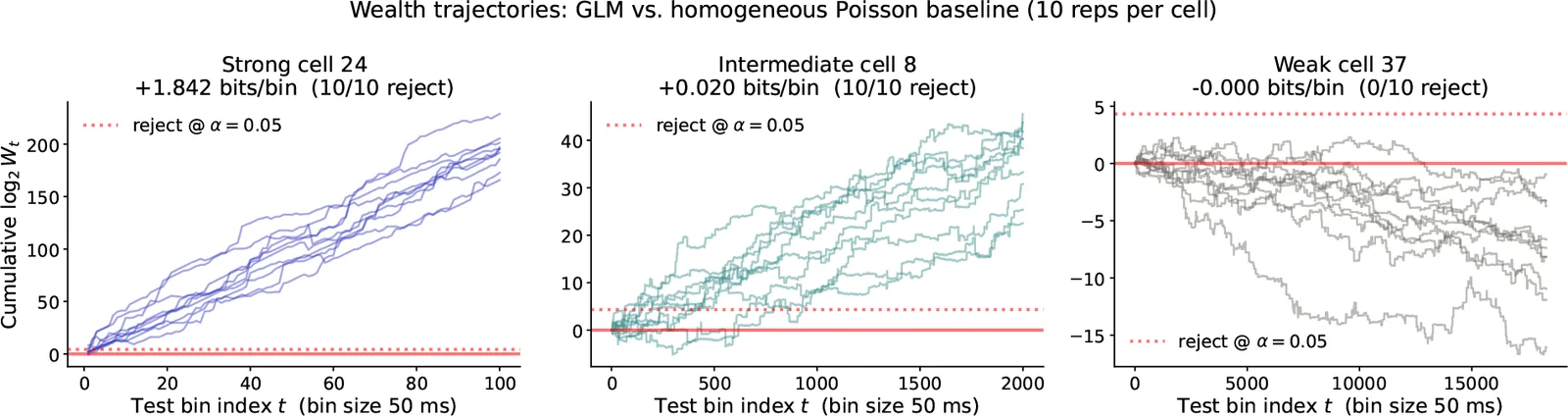
Wealth trajectories for the three example cells, ten random train/test splits each. The y-axis is on a log_2_ scale (number of doublings of the starting wealth). The dashed red horizontal line at ${log}_{2}W_{t}\approx 4.3$ marks a threshold we will motivate later. Each panel uses its own x-axis range so the dynamics of each cell are visible at an appropriate scale.

Throughout, we will use $W_{t}$ to denote the wealth of the player at round t of the game. In Figure 2, we plot how the player’s wealth $W_{t}$ evolves across the test set for each of the three cells. Every player starts with $W_{0}=1$ and the y-axis is on a log_2_ scale, so a value of ${log}_{2}W_{t}=10$ means the player has doubled their wealth ten times (a 2^10^ = 1024× return). For each cell we ran ten independent random train/test splits of the recording and overlaid all ten trajectories.^2^

Notice that the x-axis range differs across panels — the strong cell’s wealth game unfolds over 100 time bins (5 seconds), while the weak cell’s takes the full ~15 minutes of test data.

For the **strong cell** (left panel), the player’s wealth grows very fast—roughly linearly on the log_2_ scale at a rate of ~1.8 units of log wealth per round. Each round, the player nearly quadruples their wealth (on average)! The ten trajectories cluster tightly because the underlying signal is strong enough that essentially any random split of the recording produces nearly the same model and the same betting edge.

For the **intermediate cell** (middle panel) we see the same qualitative pattern — linear growth on the log_2_ scale — but at a much shallower slope of about 0.02 units of log wealth per round. This means that it takes the player 1/0.020 = 50 rounds to double their wealth, on average.

For the **weak cell** (right panel), the player has effectively no edge at all. Wealth wanders around the starting value of $W_{0}=1$ and drifts slightly *downward* on average; across all ten splits, the trajectories stay well below the dashed threshold line at the top of the panel. This is the behavior we hope to see whenever a model offers no genuine improvement over the baseline: betting on a useless predictor is, in the long run, a losing strategy.

By the end of this note, we’ll see that expected log likelihood ratios describe the rate of wealth growth. For example, $1/{ℒ}_{2}$ describes the number of rounds of betting needed (on average) to double the player’s wealth. Similarly, if we normalize by time bin size, computing $\Delta /{ℒ}_{2}$, we obtain an estimate of how much heldout data we need (in recording duration) to double the player’s wealth.

We can also connect this to a null hypothesis test with significance level α. It turns out that if the player ever generates more than 1/α units of wealth, we can reject the null hypothesis that the true distribution, P, is equal to the baseline model, B, with type-I error rate less than α. For example, if we set α=0.05, then we can reject the null hypothesis if we ever obtain $W_{t}>1/\alpha =20$.

**Figure 3.**
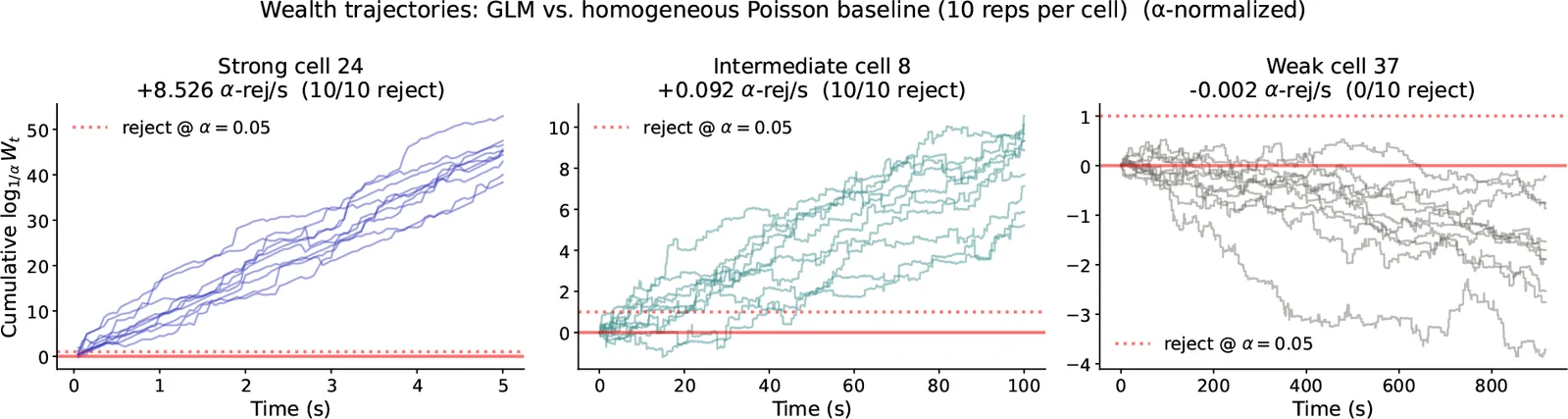
The same wealth trajectories as Figure 2, now plotted in units of ${log}_{1/\alpha }W_{t}$ with α=0.05. The dashed red line at y=1 marks the wealth required to reject the null at level α. The slope of each trajectory is the rate at which the player accumulates rejection-worth of evidence.

In light of this, the expected base-(1/α) log likelihood ratio, denoted ${ℒ}_{1/\alpha }$, tells us the rate at which we accumulate evidence to reject the null hypothesis that P=B. Similarly, $1/{ℒ}_{1/\alpha }$ and $\Delta /{ℒ}_{1/\alpha }$ respectively tell us how much data we need (on average) to reject the null in units of time bins and seconds. Figure 3 below re-plots the data from Figure 2 on this log scale. Further, the x-axis is plotted in units of recorded time (by dividing by time bin size Δ). The units of the y-axis now correspond to the number of times the player has accumulated enough wealth to reject the null hypothesis at significance level α. For the strong cell (*left*) that timescale is around 120 ms; for the intermediate cell it is around 11 seconds; for the weak cell the trajectories never cross the threshold, and we therefore fail to reject the null.

A common alternative normalization is *bits per spike*, the metric mentioned at the top of the post. If ${\overset{‾}{\lambda }}_{b}$ is the baseline’s expected spike count per bin, then $bits/spike={ℒ}_{2}/{\overset{‾}{\lambda }}_{b}$ coincides with the player’s wealth growth per spike observed, rather than per bin elapsed. Normalizing by the number of spikes is potentially useful when comparing models fit to neurons with very different firing rates. However, since the betting game is naturally indexed by time bins of the recording, the rest of the post will stick to normalizing the log likelihood ratio in units of time.

Now that we’ve sketched some results, it’s time to make this picture rigorous. Specifically, we want to (1) define exactly how each round of betting works, (2) explain the optimal strategy that player Q can implement, and (3) understand what the significance threshold line in the figure means in terms of a formal hypothesis test.

## 3 Introducing the Game

The game starts by giving player Q one unit of wealth:

$$W_{0}=1$$

At each round of the game, player Q uses all of their wealth to purchase *prediction contracts*, specified by a function C(x)>0. On each round, indexed by t, we sample an observation $X_{t}∼P$ and pay the player $C\left(X_{t}\right)$ units of wealth. Thus, the player receives a random sequence of returns $C\left(X_{1}\right),C\left(X_{2}\right),C\left(X_{3}\right),\ldots$ over discrete rounds of the game. The wealth updates according to:

(3) $$W_{t}=\left(W_{t-1}/\pi (C)\right)\cdot C\left(X_{t}\right).$$

where π(C) denotes the *price* of the contract.

Equation (3) is simple. The first term, $W_{t-1}/\pi (C)$, is the *number of contracts* the player is able to purchase given their previous wealth at round t-1. The second term, $C\left(X_{t}\right)$, is the payoff of each contract. Note that we allow the wealth and the number of purchased contracts to be infinitely divisible into fractions.

Intuitively, the price of a contract is set by what people are willing to buy and sell it for, which reflects their expectations about the underlying distribution P. For the purposes of our game we’ll assume that the market consensus—or the “wisdom of the crowd”—coincides with the baseline model B. Formally, it turns out that the fair price of a contract is given by its expected value under the market consensus distribution. That is,

(4) $$\pi (C)=E_{X∼B}\;[C(X)].$$

See Supplementary Note 1 for a quick derivation of this equation.

The pricing constraint in equation (4) allows us to simplify the structure of the game by assuming that the contracts have unit price. Indeed, for any contract C(⋅), we can define a new contract S(x)=C(x)/π(C) which has unit price, π(S)=1. The wealth update for C and S is equivalent since

$$\left(W_{t-1}/\pi (C)\right)\cdot C\left(X_{t}\right)=W_{t-1}\cdot S\left(X_{t}\right),$$

by the definition of S. Therefore, for the rest of this note we will focus on the simplified wealth process

### Simplified wealth process.

Assuming that the player purchases positive contracts S(x)>0 of unit price, i.e.

(5) $$E_{X∼B}[S(X)]=1$$

then the player’s wealth evolves according to

(6) $$W_{t}=W_{t-1}\cdot S\left(X_{t}\right).$$

## 4 Choosing the optimal contract function

Player Q is allowed to choose the function S(⋅) however they like, so long as it satisfies (5). Out of this space of feasible contracts, which one should Q choose to play?

After T rounds of betting according to equation (6), the player will accumulate

(7) $$W_{T}=\prod_{t=1}^{T}S\left(X_{t}\right)=exp\;log\prod_{t=1}^{T}S\left(X_{t}\right)$$

(8) $$=exp\sum_{t=1}^{T}logS\left(X_{t}\right)$$

(9) $$=exp\left(T\cdot \left(\frac{1}{T}\sum_{t=1}^{T}log\;S\left(X_{t}\right)\right)\right)$$

(10) $$\approx exp(T\cdot E_{X∼P}\;log\;S(X))$$

units of wealth. The approximation in the final line comes from replacing the empirical expectation $\frac{1}{T}\sum_{t=1}^{T}log\;S\left(X_{t}\right)$ with the true expected value under P.

Since the player believes that P=Q, they anticipate that their wealth can grow exponentially over time according to:

(11) $$W_{T}\approx exp\left(T\cdot E_{X∼Q}\;log\;S(X)\right)$$

To maximize their rate of wealth growth, a reasonable strategy is to choose S(⋅) in order to

(12) $$\text{maximize}\;E_{X∼Q}\;log\;S(X)\;\text{subject}\;\text{to}\;(5)$$

Quite pleasingly, as shown in Supplementary Note 2, the solution to this optimization problem, denoted $S^{⋆}$, turns out to be the likelihood ratio!

(13) $$S^{⋆}(x)=\frac{q(x)}{b(x)}$$

Combining equations (13) and (10) with the definition of ℒ in (1), we see that the long-run wealth of the player is approximated by

(14) $$W_{T}\approx exp\;(ℒ\cdot T)$$

for large T. In other words, the wealth accumulated by player Q in the game will, over the long run, grow or decay exponentially fast at a rate given by the expected log-likelihood ratio.

## 5 Some interpretations

We are now in a position to rigorously reinterpret Figure 2. Taking logarithms on both sides of (14) and changing to base-2 yields a linear relationship between the log-wealth and time bin index:

(15) $${log}_{2}\left(W_{T}\right)\approx {ℒ}_{2}\cdot T.$$

The reciprocal $1/{ℒ}_{2}$ is therefore the average *doubling time* of the wealth process in units of time bins (or equivalently, rounds of betting). Multiplying by the time bin size, $\Delta /{ℒ}_{2}$, changes the units of the doubling time to seconds of recording. For example, in Figure 2, the strong cell (*left*) doubles its wealth every ~25 ms while the intermediate cell (*middle*) doubles its wealth every ~2.5 seconds.

It is also interesting to note a connection to the KL divergence. While ℒ determines the *actual* rate of wealth growth, the player’s anticipated rate of wealth growth is given by replacing P in (10) with Q, yielding:

(16) $$E_{X∼Q}[log\;q(X)/b(X)]$$

which is precisely the KL divergence, $D_{KL}(Q\;\|\;B)$.^3^

**Figure 4.**
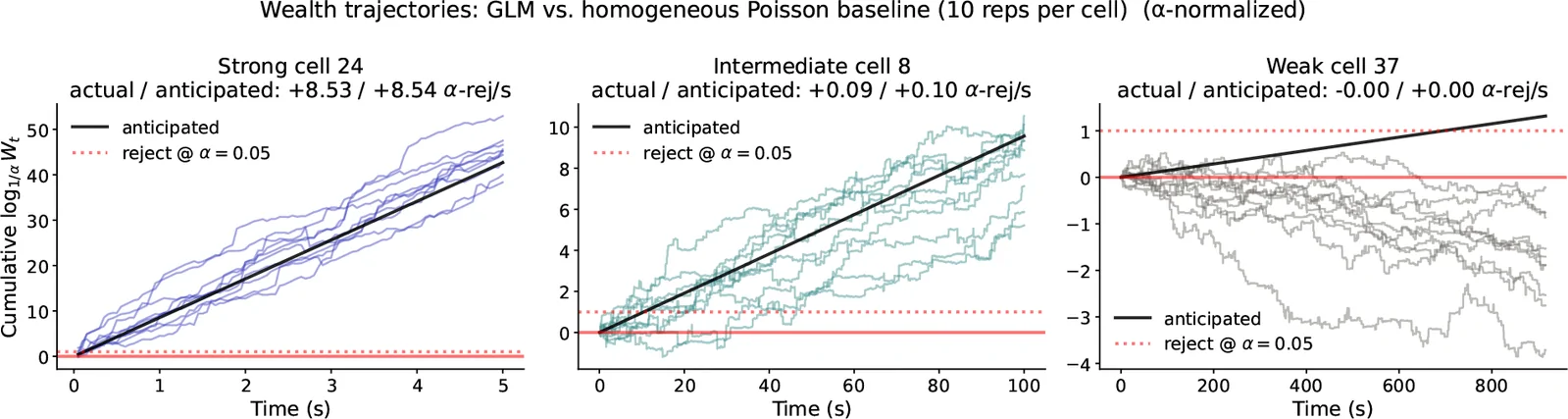
Same wealth trajectories as Figure 3, now with the player’s anticipated rate of wealth growth overlaid as a black line. The slope of the line is $D_{KL}(Q\;\|\;B)$ per round of betting, averaged across the ten train/test splits. For the strong and intermediate cells, the anticipated and realized trajectories agree closely. For the weak cell, the anticipated line drifts slightly above zero while the realized trajectories drift slightly below — a small but real signature of overfitting on the training data.

Figure 4 shows the same wealth trajectories as in Figure 3 with the expected rate of wealth growth, given by $D_{KL}(Q\;\|\;B)$, overlaid as a dark black line. For the strong and intermediate cells (*left* and *middle* panels), the anticipated and realized growth rates agree, indicating that the GLM model is well-calibrated on this dataset. For the weak cell, the anticipated line climbs gradually above zero while the realized trajectories drift slightly below. This is suggestive of overfitting: Q believes it has a tiny edge that doesn’t actually exist on heldout data.

## 6 Connection to Hypothesis Testing

We have not yet drawn a rigorous connection to null hypothesis testing, as promised by the plots in Figures 3 and 4. The connection is simple and follows concretely from a result called Ville’s inequality (Ville 1939; Howard et al. 2020), summarized below.

### Ville’s Inequality (informal).

Let ${\left(M_{t}\right)}_{t\geq 0}$ be a sequence of random variables such that $M_{t}\geq 0$ almost surely for all t and $E\left[M_{t}|M_{0},\ldots ,M_{t-1}\right]\leq M_{t-1}$ for all t. Then for any α>0,

$$Pr\left(\underset{t\geq 0}{sup}\;M_{t}\geq 1/\alpha \right)\leq \alpha \cdot E\left[M_{0}\right].$$

Intuitively, this result states that if each step of the random sequence ${\left(M_{t}\right)}_{t\geq 0}$ is not increasing in expectation,^4^ then the probability of ${\left(M_{t}\right)}_{t\geq 0}$ *ever* crossing above a high threshold is small, even if we run the simulation infinitely far into the future. Supplementary Note 3 sketches a quick proof of Ville’s inequality.

To see why this matters in our setting, suppose that the baseline B is in fact the true data-generating distribution—i.e. P=B. Then we would have

$$E_{X_{t}∼B}\left[W_{t}|W_{0}\ldots W_{t-1}\right]=W_{t-1}\cdot E_{X_{t}∼B}\left[\frac{q\left(X_{t}\right)}{b\left(X_{t}\right)}\right]=W_{t-1}.$$

since the expectation of the likelihood ratio equals one.^5^ This means that we can apply Ville’s inequality, which tells us that the probability of player Q’s wealth *ever* exceeding the threshold 1/α—at any round of the game, even infinitely far into the future—is at most α:

$$P\left(\underset{t\geq 0}{sup}\;W_{t}\geq 1/\alpha \right)\leq \alpha .$$

This furnishes an *anytime-valid* hypothesis test (Ramdas et al. 2023) of the null $H_{0}:P=B$. We may reject $H_{0}$ at level α as soon as $W_{t}$ crosses 1/α, regardless of how many rounds have been played. Unlike classical fixed-sample tests, we are free to peek at the data, stop early, or keep collecting more samples, all without inflating the type I error rate. For example, if we use α=0.05 (as is customary), then we can reject the null hypothesis that P=B if player Q’s wealth *ever* exceeds 20.

## 7 Take Home Message

A central message of this note is that the expected log-likelihood ratio, ℒ, can be interpreted as the exponential rate at which a player generates wealth in a betting game based on forecasting future events. For example, log(2)/ℒ defines the time it takes for the player to double their wealth (in units of time bins).

The betting game interpretation is satisfying, but it involves some effort to conceptualize and formally derive. In an effort to simplify the take home message, I propose two main summary statistics: the **samples to significance**, τ, and the **time to significance**, given by τΔ.

### Time to Significance.

Let ℒ>0 denote the expected log-likelihood ratio of a model Q relative to baseline B, as in equation (1). The *samples to significance*,

$$\tau =\frac{-log(\alpha )}{ℒ},$$

reflects the number of heldout samples needed on average to reject the null hypothesis $H_{0}\;:\;P=B$ at significance level α. If each sample is a time bin of Δ seconds, the corresponding *time to significance* is given by τΔ=-Δlog(α)/ℒ.

In practice, we estimate these quanitites by first fitting Q and B on training data and using heldout test data to compute an estimate of the expected log-likelihood ratio, $\hat{ℒ}$ in (2). The time to significance is a summary statistic of model performance that can be used in place of, or complementary to, the conventional “bits per spike” metric. This gives τΔ≈120ms for the strong example cell and τΔ≈11s for the intermediate example cell in the figures. For the weak example cell, τ is undefined since $\hat{ℒ}<0$, and so the signifiance threshold is never crossed in expectation.

Because τ is a strictly decreasing function of ℒ, it ranks models identically to the heldout log-likelihood (and hence to bits per spike). Thus, it is not a fundamentally new statistic, but a more interpretable *unit* for an existing one, answering the question: “how much data do I need to confirm that Q beats B?” Quantities similar to the time to significance appear in prior literature, notably Wald’s sequential probability ratio test (Wald 1945).

## 8 Limitations

The material in this note comes with at least three caveats.

### Variability across splits.

The trajectories in Figures 2–4 come from ten independent train/test splits, so part of their spread reflects variation in the *fitted* model Q rather than variation in the betting game itself. The game as formulated conditions on a fixed Q; reporting a distribution of τ over splits is a practical hedge, not a confidence interval for τ.

### Independence and temporal structure.

The derivation assumes heldout samples are i.i.d., which we enforced by shuffling time bins before splitting. That discards the substantial autocorrelation present in neural recordings. For models whose advantage over a baseline lies precisely in capturing temporal dependence — spike-history filters, latent dynamics — shuffling would misstate the evidence. The martingale argument itself does not require independence, only that contracts be priced under the conditional distribution implied by the baseline given the past; extending the demonstration to sequentially conditioned baselines a natural extension.

### Composite nulls.

The null treated here, $H_{0}:P=B$, is simple: it fixes a single baseline distribution. In practice B is itself fit to training data, so a more honest null would be composite—e.g., the family of all homogeneous Poisson processes. Because B is estimated on training data independent of the test set, the simple-null treatment is a reasonable approximation, but the distinction could matter for small training sets and for more expressive baselines. See Wasserman, Ramdas, and Balakrishnan (2020), Grünwald, de Heide, and Koolen (2024), and the monograph by Ramdas and Wang (2025) for more rigorous treatment of composite nulls.

## Supplementary Material

Supplement 1

## Data Availability

The analysis script that produced Figures 1–4, together with the sources for this document, are available at https://github.com/neurostatsblog/neurostatsblog.github.io under code/betting/ and content/. The recordings are the publicly available mouse anterior-thalamus head-direction dataset of Peyrache et al. (2015), downloaded through the example-data facility of **nemos**.

## Footnotes

1 It is possible to make the math work without making this assumption. Generally, this would let us turn “> relations” into “≥ relations” and likewise turn “≤ relations” into “< relations”. However, for simplicity we just stick to strict inequalities for the purpose of this note.

2 It is worth pointing out an important approximation. We use ten independently generated train and test splits to generate each random wealth trajectory. Thus, we fit ten different GLM models and have them “play the betting game” on ten different heldout test sets. Thus, some of the variability in the trajectories is due to differences in the training data. Our exposition of the betting game does not account for this. Another approximation worth noting is that we do not account for autocorrelation in the observations—we shuffle the order of time bins on each split. Extending the betting game to time series data with autocorrelation could be important for comparing certain models of neural data.

3 This interpretation of KL divergence is due to JL Kelly Jr. in a 1956 tech report (Kelly 1956). The principle that the player should choose their bet to maximize the term in the exponent appearing in (10) is named after him—it is known as the Kelly criterion in quantitative finance.

4 For those who appreciate jargon, we call ${\left(M_{t}\right)}_{t\geq 0}$ a *supermartingale* if it satisfies $E\left[M_{t}|M_{0},\ldots ,M_{t-1}\right]\leq M_{t-1}$. In the stricter case where the inequality is saturated, i.e. $E\left[M_{t}|M_{0},\ldots ,M_{t-1}\right]=M_{t-1}$, we call ${\left(M_{t}\right)}_{t\geq 0}$ a *martingale*. Ville’s inequality says that all nonnegative supermartingales (and martingales) are upper bounded for all time with high probability.

5 Concretely, $E_{X∼B}\left[\frac{q(X)}{b(X)}\right]=\int \;\frac{q(x)b(x)}{b(x)}dx=\int \;q(x)dx=1$.
