# Supplementary material for: Bits per Spike as a Betting Game: An interpretable unit for held-out log-likelihood in neural data analysis
Source: ArXiv. 2026 Jul 30:arXiv:2607.28779v1. Preprint. [Version 1] (PMC13484423)
Supplement: Supplementary file 1 [file NIHPP2607.28779v1-supplement-1.pdf]

A Supplementary Note 1

Here we sketch how the market price constraint (4) arises in more detail. For simplicity, let's consider a case where the random outcomes $X \sim P$ take on one of n discrete values. That is, $X \in \{1, \ldots, n\}$ almost surely. The same argument can be extended to continuous-valued random variables with sufficient care.

By assuming there are only n discrete outcomes, then we can express any potential contract function $C(\cdot)$ as a finite linear combination of elementary basis functions:

$$C(x) = \sum_{i=1}^{n} r_i \delta_i(x) \tag{17}$$

where $r_1, \ldots, r_n$ are scalar coefficients denoting the return of outcome $x = i$,

$$r_i = C(i) \ , \tag{18}$$

and $\delta_1, \ldots, \delta_n$ are contracts that pay off one unit of wealth if the outcome is $x = i$,

$$\delta_i(x) = \begin{cases} 1 & x = i \\ 0 & x \neq i \end{cases} .$$

Recall that our goal is to show $\pi(C) = \mathbb{E}\left[C(X)\right]$, with the expectation taken with respect to some appropriate choice of distribution B. We will show that this is true if the market is organized such that no player can receive "free money" without taking any risk (in other words, there are no arbitrage opportunities). We assume that any player can either buy or sell contracts, and that fractional contracts are supported in the market (e.g. by sharing contracts with other players).

Observation 1 – The pricing function π must be linear. For any scalar $a > 0$, we must have $\pi(a \cdot C) = a\pi(C)$. If $\pi(a \cdot C) > a \cdot \pi(C)$ a player would get free money by selling the contract $a \cdot C$ and simultaneously buying a contracts of C. For any outcome $X \sim P$, the player neither gains nor loses any wealth and they pocket $\pi(a \cdot C) - a \cdot \pi(C) > 0$ units of wealth. Conversely, if $\pi(a \cdot C) < a \cdot \pi(C)$ a player would get free money by buying the contract $a \cdot C$ and simultaneously selling a contracts of C. An arbitrage-free pricing scheme therefore must satisfy $\pi(a \cdot C) = a\pi(C)$.

Next, for any two contracts C_1 and C_2 we must have that $\pi(C_1 + C_2) = \pi(C_1) + \pi(C_2)$. The argument is quite similar to above. If $\pi(C_1 + C_2) > \pi(C_1) + \pi(C_2)$ then a player would get free money by selling the contract $C_3(x) = C_1(x) + C_2(x)$ and simultaneously buying C_1 and C_2. Since $C_1(x) + C_2(x) - C_3(x) = 0$ for all x, the player neither gains nor loses any wealth based on the random outcome and they pocket $\pi(C_3) - \pi(C_1 + C_2) > 0$ units of wealth. Conversely, if $\pi(C_1 + C_2) < \pi(C_1) + \pi(C_2)$ then run the same trade in reverse to get free money.

Taken together, we conclude that pricing must be linear. Applying this to (17) we can conclude that the price of any contract can be written down as:

$$\pi(C) = \sum_{i=1}^{n} r_i \pi(\delta_i) \tag{19}$$

Thus, we can determine the price of any contract by determining the prices of the elementary basis contracts $\delta_1, \ldots, \delta_n$.

Observation 2 – the prices $\pi(\delta_1), \ldots, \pi(\delta_n)$ are nonnegative and sum to one. First we prove nonnegativity. The random payout for each contract, $\delta_i(X)$ for $X \sim P$, is greater than or equal to zero almost surely. Thus, the price of each contract must be nonnegative—if it were

negative, it would imply that the player is *paid* to accept a contract that never results in a loss (i.e. receive free money).

Next we prove the normalization condition that $\sum_i \pi(\delta_i) = 1$. Note that we can construct a contract with constant payoff $C(X) = 1$, almost surely, by setting $r_1 = r_2 = \cdots = r_n = 1$ in equation (17). If the price of this contract were less than one, a player would get free money by purchasing it. Likewise, if the price were greater than one, a player would get free money by selling it.

Putting it together. From observation 2, it is clear that the prices $\pi(\delta_1), \dots, \pi(\delta_n)$ define a probability measure over outcomes $x \in \{1, \dots, n\}$. Call this probability measure B. Then, recalling that $r_i = C(i)$ denotes the return of outcome $x = i$ under the contract, we deduce from equation (19):

$$\pi(C) = \sum_{i=1}^{n} r_i \pi(\delta_i) = \mathbb{E}_{X \sim B}\left[C(X)\right]$$

confirming our claim that the price of a contract is given by the expected payoff of the contract under an appropriate distribution B.

B Supplementary Note 2

We prove that the optimization problem stated in (12) is solved by the likelihood ratio $S^\star(x) = q(x)/b(x)$ given in (13).

The argument relies on a useful reparameterization. Let $r(x) = b(x)\, S(x)$ and note that $r(x)$ is a probability density. Indeed, $r(x) > 0$ since both b and S are strictly positive, and

$$\int r(x)\, dx \;=\; \int b(x)\, S(x)\, dx \;=\; \mathbb{E}_{X\sim B}[S(X)] \;=\; \pi(S) \;=\; 1$$

by the unit-price constraint (5). Conversely, since we assumed $b(x) > 0$ everywhere on the support of P, any probability density r defines a feasible contract function via $S(x) = r(x)/b(x)$.

Now substitute $S(x) = r(x)/b(x)$ into the objective in (12), then add and subtract $\mathbb{E}_{X\sim Q} \log q(X)$:

$$\mathbb{E}_{X\sim Q} \log S(X) \;=\; \mathbb{E}_{X\sim Q} \log \frac{r(X)}{b(X)} \tag{20}$$

$$\;=\; \mathbb{E}_{X\sim Q} \log \frac{q(X)}{b(X)} \;-\; \mathbb{E}_{X\sim Q} \log \frac{q(X)}{r(X)} \tag{21}$$

$$\;=\; D_{\mathrm{KL}}(Q \,\|\, B) \;-\; D_{\mathrm{KL}}(Q \,\|\, R). \tag{22}$$

The first term, $D_{\mathrm{KL}}(Q \,\|\, B)$, does not depend on the player's choice of contract. The second term, $D_{\mathrm{KL}}(Q \,\|\, R)$, is non-negative by Gibbs' inequality, with equality if and only if $R = Q$. To maximize the objective, the player should therefore choose $R = Q$, that is, $r(x) = q(x)$. Translating back to a contract function via $S = r/b$ yields

$$S^\star(x) \;=\; \frac{q(x)}{b(x)},$$

as claimed in (13). The maximum achievable value of the objective is $D_{\mathrm{KL}}(Q \,\|\, B)$ — the exponential rate at which the player anticipates their wealth will grow, recovering the Kelly-criterion interpretation discussed in the main text.

C Supplementary Note 3

Here we give an informal proof sketch of Ville's inequality. The remarkable part of this result is that the inequality holds *uniformly over all time t*. So if we simulated a very large number of wealth trajectories (i.e. player Q gets to restart and play the game many times), then only a small fraction, α, of the trajectories would ever cross above $1/\alpha$, *even if each individual trajectory were simulated infinitely long.*

Recall that we are given a discrete-time sequence $(M_t)_{t \geq 0}$, satisfying $\mathbb{E}[M_t \mid M_0, \ldots, M_{t-1}] \leq M_{t-1}$ and $M_t \geq 0$ for all t. A sequence with these two properties is called a *nonnegative supermartingale.*

Let c denote the first time that the trajectory crosses above a threshold $\lambda > 0$. That is, c is the smallest natural number such that $M_c \geq \lambda$. If the trajectory *never* crosses above λ, we take this as meaning $c = \infty$. Our goal is to prove

$$\Pr(c \text{ is finite}) \leq \frac{\mathbb{E}[M_0]}{\lambda} \tag{23}$$

which is more-or-less identical to the result we want. In the main post, we chose the threshold to be $\lambda = 1/\alpha$.

The trick is to define a new *nonnegative supermartingale* $(Z_t)_{t \geq 0}$ as

$$Z_t = \begin{cases} M_t & \text{if } t < c \\ \lambda & \text{if } t \geq c \end{cases} \tag{24}$$

Note that if $c = \infty$, then M_t never crosses above the threshold and Z_t is just a copy of M_t for all t. It is easy to show that if $(M_t)_{t \geq 0}$ is a nonnegative supermartingale, then so is $(Z_t)_{t \geq 0}$ (exercise to the reader).

Now for any fixed value of t, we can apply Markov's inequality to conclude that

$$\Pr(c \leq t) = \Pr(Z_t \geq \lambda) \leq \frac{\mathbb{E}[Z_t]}{\lambda} \leq \frac{\mathbb{E}[Z_0]}{\lambda} \tag{25}$$

The final inequality follows from the fact that $(Z_t)_{t \geq 0}$ is a nonnegative supermartingale, which implies $\mathbb{E}[Z_t] \leq \mathbb{E}[Z_0]$.

Further, $\mathbb{E}[Z_0] \leq \mathbb{E}[M_0]$, since Z_t is a clipped version of M_t. Thus, we have:

$$\Pr(c \leq t) \leq \frac{\mathbb{E}[M_0]}{\lambda}. \tag{26}$$

Notice that the right hand side is not a function of t. There is a way to rigorously take the limit of $t \to \infty$ on both sides of the inequality to yield our desired result, equation (23).
